# Single intravenous administration of oncolytic adenovirus TILT-123 results in systemic tumor transduction and immune response in patients with advanced solid tumors

**DOI:** 10.1186/s13046-024-03219-0

**Published:** 2024-11-06

**Authors:** Elise Jirovec, Dafne C. A. Quixabeira, James H. A. Clubb, Santeri A. Pakola, Tatiana Kudling, Victor Arias, Lyna Haybout, Katriina Jalkanen, Tuomo Alanko, Tine Monberg, Amir Khammari, Brigitte Dreno, Inge Marie Svane, Matthew S. Block, Daniel A. Adamo, Johanna Mäenpää, Claudia Kistler, Suvi Sorsa, Otto Hemminki, Anna Kanerva, João M. Santos, Victor Cervera-Carrascon, Akseli Hemminki

**Affiliations:** 1 Cancer Gene Therapy Group, Translational Immunology Research Program, University of Helsinki, Helsinki, Finland; 2 TILT Biotherapeutics Ltd, Helsinki, Finland; 3 Comprehensive Cancer Center, Helsinki University Hospital, Helsinki, Finland; 4 National Center for Cancer Immune Therapy (CCIT-DK), Department of Oncology, Copenhagen University Hospital, Herlev, Denmark; 5 Department of Dermatology, Nantes University, CHU Nantes, CIC1413, INSERM, CNRS, Immunology and New Concepts in ImmunoTherapy, INCIT, UMR 1302, Nantes, France; 6 Nantes University, INSERM, CNRS, Immunology and New Concepts in ImmunoTherapy, INCIT, UMR 1302, Nantes, France; 7 Mayo Clinic Cancer Center, Minnesota, Rochester, USA; 8 Docrates Cancer Center, Helsinki, Finland; 9 Faculty of Medicine and Medical Technology, and Cancer Center, Tampere University and University Hospital, Tampere, Finland; 10 Department of Urology, Helsinki University Hospital, Helsinki, Finland; 11 Department of Gynecology and Obstetrics, Helsinki University Hospital, Helsinki, Finland

**Keywords:** Oncolytic Virus, TILT-123, Intravenous Delivery, Adenovirus, Solid Tumors, Immunotherapy

## Abstract

**Background:**

A limitation of approved oncolytic viruses is their requirement for intratumoral (i.t.) injection. TILT-123 (igrelimogene litadenorepvec, Ad5/3-E2F-D24-hTNFα-IRES-hIL-2) is a chimeric oncolytic adenovirus suitable for intravenous (i.v.) delivery due to its capsid modification and dual selectivity devices. It is armed with tumor necrosis alpha and interleukin-2 for promoting T-cell activation and lymphocyte trafficking to tumors, thereby enhancing the antitumor immune response. Here, we present the findings after a single i.v. administration of TILT-123 in three phase I dose escalation clinical trials.

**Methods:**

Patients with advanced solid tumors initially received a single i.v. dose of TILT-123 ranging from 3 × 10^9^ to 4 × 10^12^ viral particles (VP). Blood was collected at baseline, 1, 16, and 192 h (7 days) post-treatment for bioavailability and serum analysis. Tumor biopsies were collected prior to treatment and 7 days post-treatment for analysis of viral presence and immunological effects. Patients did not receive any other cancer therapies during this period.

**Results:**

Across all three trials (TUNIMO, TUNINTIL, and PROTA), 52 total patients were treated with i.v. TILT-123. Overall, TILT-123 was found to be well-tolerated, with no dose-limiting toxicities observed. Post-treatment tumor biopsies showed expression of viral genes, presence of TILT-123 adenovirus proteins or DNA, and changes in immune cell infiltration from baseline. Increased virus dose did not lead to increased virus detection in tumors. Median overall survival was longer in patients with confirmed presence of TILT-123 in post-treatment biopsies (280 versus 190 days, *p* = 0.0405).

**Conclusion:**

TILT-123 demonstrated safety and significant intratumoral immunomodulation following a single i.v. administration, warranting further investigation.

**Trial registrations:**

TUNIMO—NCT04695327. Registered 4 January 2021, https://clinicaltrials.gov/study/NCT04695327. TUNINTIL—NCT04217473. Registered 19 December 2019, https://clinicaltrials.gov/study/NCT04217473. PROTA—NCT05271318. Registered 4 February 2022, https://clinicaltrials.gov/study/NCT05271318.

**Supplementary Information:**

The online version contains supplementary material available at 10.1186/s13046-024-03219-0.

## Background

Solid tumors account for 90% of all cancers and are typically managed using treatment methods that include surgical resection, chemotherapy, biological, hormonal, targeted therapies, immunotherapy, and radiotherapy [1]. However, these standard treatments may not be sufficient for patients with advanced-stage disease, who often do not achieve a curative response, as illustrated by a 5-year survival rate of 50% in patients with advanced ovarian cancer [2]. Other solid tumors such as pancreatic, lung, and gastrointestinal cancers face similar challenges, with late diagnoses and limited curative treatments [3–5]. These patients have few effective treatment options, as current therapies are often not curative, and frequently cause adverse side effects, highlighting the need for innovative and more effective therapeutic strategies [6].

While immunotherapies such as checkpoint inhibitors have made their breakthrough in the treatment of solid tumors, typically only a minority of patients respond to this therapy [7]. For instance, advanced melanoma patients treated with anti-PD-1 have shown an objective response rate of 20%, likely due to complex mechanisms of immunotherapy resistance such as altered tumor microenvironment (TME) metabolism and immunosuppression [8, 9]. Therefore, finding innovative strategies to overcome the inhibitory TME and enhance the efficacy of immunotherapy is of great interest.

One promising approach is the use of oncolytic viruses (OVs). OVs have emerged as potent immunogenic agents that are able to harness the immune system to selectively target cancer cells and promote antitumor immunity [10]. OV-mediated oncolysis activates the immune system by inducing immunogenic cell death, the release of pathogen-associated molecular patterns (PAMPs), danger-associated molecular patterns (DAMPs), tumor antigens, and the release of new viral particles that can infect neighboring tumor cells [11].

TILT-123 (Ad5/3-E2F-D24-hTNFα-IRES-hIL-2, igrelimogene litadenorepvec) is a human 5/3 chimeric adenovirus optimized for intravenous delivery and generation of a potent T-cell mediated antitumor response [12–14]. The capsid modification (5/3 chimerism) enhances tumor tropism while dual selectivity devices allow high systemic doses. Serotype chimerism helps avoid neutralization by pre-existing antibodies, as the chimeric virus does not exist in nature, ensuring no prior immune exposure to this specific form [15]. The mechanism of action of TILT-123 has been characterized in previous pre-clinical studies. Briefly, TILT-123 exhibits oncolytic activity by lysing tumor cells, which subsequently activates antitumor immunity through DAMP and PAMP signaling [14, 16]. Additionally, TILT-123 is armed with two immunostimulatory transgenes, interleukin-2 (IL-2) and tumor necrosis factor alpha (TNFα). The expression of these transgenes is tightly coupled to viral replication, ensuring that transgenes are expressed specifically within tumors and released into the local tumor environment upon tumor lysis [12]. These transgenes facilitate immune T cell trafficking, activation, proliferation, and induction of tumor cell death, thereby enhancing the overall antitumor immune response [13, 14]. The selection of the transgenes (Il-2 and TNFα) involved a detailed comparison of secretory molecules with known effects on T-cell activation and recruitment, optimizing the therapeutic potential of the virus [13].

The field of oncolytic virotherapy has seen many advancements, with numerous OVs being tested in clinical settings, and notably the approval of oncolytic herpes simplex virus encoding granulocyte–macrophage colony-stimulating factor (talimogene laherparepvec) by the Food and Drug Administration (FDA) and European Medicines Agency (EMA) in 2015. Other approved OVs include Oncorine in China and Delytact in Japan [17, 18]. However, clinical research on OVs is predominantly focused on intratumoral administration, and in fact, all approved OVs rely on this approach [19]. In theory, i.t. delivery allows for a high viral load to be administered directly into the tumor, reducing systemic viral clearance by antiviral homeostatic mechanisms such as liver clearance and neutralizing antibodies (NAbs) [20]. However, i.t. injections can be technically challenging, are limited to accessible tumors, and may cause localized side effects such as inflammation and pain at the injection site [21, 22]. The complexity of i.t. injection, which can be challenging to perform at community oncology practices and smaller hospitals, may explain why approved OVs are not frequently used in routine clinical practice.

In contrast, i.v. delivery of OVs provides systemic distribution, making it advantageous for targeting metastatic sites or hard-to-reach tumors, and offering a more convenient method of administration which might be more attractive in a routine clinical setting [21]. Of note, so far i.v. delivery has not been successful enough in OV clinical trials to merit regulatory approval, and it remains a less common approach than local delivery. A comparative analysis of ongoing clinical trials emphasizes this trend: a ClinicalTrials.gov search on 9 June 2024 of ongoing OV trials found 71 trials investigating i.t. delivery, compared to 24 trials investigating i.v. delivery. This background data highlights the need for further investigation into the safety and efficacy of i.v. administration of OVs in the clinical setting. Here we report data on the i.v. use of TILT-123 in three separate phase I dose escalation trials- TUNIMO, TUNINTIL, and PROTA, across multiple solid tumor types. We focus on the bioavailability, biological, and overall survival effects of a single i.v. administration of TILT-123.

## Materials & Methods

### Patients

Twenty eligible patients with various solid tumor types were enrolled in TUNIMO (NCT04695327). Inclusion and exclusion criteria have been previously described [23].

Seventeen eligible patients diagnosed with melanoma were enrolled in TUNINTIL (NCT04217473). Inclusion criteria for this trial included patient age between 18–75 years, pathologically confirmed refractory and recurrent melanoma with no available therapies, at least one prior line of medical treatment, tumor diameter of > 14 mm without signs of necrosis, at least one additional tumor for local injections, eligibility for adoptive T-cell therapy, adequate hepatic (bilirubin < 1–5 × upper limit of normal (ULN), AST and ALT < 3 × ULN), cardiac (platelets > 75 000 mm^3^, hemoglobin ≥ 100 g/L and renal functions (GFR > 60 mL/min). Key exclusion criteria included use of immunosuppressive medications, any anti-cancer therapy 30 days prior to enrolment, uncontrolled cardiac or vascular disease, hepatic dysfunction, and previous treatment with oncolytic adenovirus (administered i.t.) or previous adoptive cell therapy treatment.

Fifteen eligible patients diagnosed with platinum-resistant or refractory ovarian cancer were enrolled in PROTA phase 1a (NCT05271318). Inclusion criteria for this trial included minimum patient age of 18 years, histologically confirmed resistant or platinum-based chemotherapy refractory ovarian cancer, life expectancy longer than 3 months, at least one tumor > 14 mm in diameter or carcinomatosis for local virus injection, ECOG/WHO performance score of 0–1 at screening, adequate hepatic (total bilirubin < 1.5 × ULN, AST and ALT ≤ 2.5 × ULN or ≤ 5 × ULN for patients with liver metastases), cardiac and renal functions (GFR > 45 mL/min). Key exclusion criteria included autoimmune disease requiring systemic treatment, prior treatment with immune checkpoint inhibitors (PD-1, PD-L1 or PD-L2) and discontinuation due to Grade 3 or higher immune-related adverse events, uncontrolled cardiac or vascular disease, and hepatic dysfunction.

## Treatment

On day 1 of each trial, patients were treated with a single i.v. injection of TILT-123 resuspended in 0.9% saline in a volume of 10 – 40 mL. TILT-123 or Ad5/3-E2F-D24-hTNFα-IRES-hIL-2 was constructed and tested preclinically as previously described [14]. The transgenes (hTNFα and hIL-2) were placed into the E3 region of the virus. The encoded dual selectivity devices, the E2F promoter and a 24-base pair deletion in the constant region 2 of E1A, render the virus selective for tumor cells defective in the retinoblastoma/p16 pathway, a universal phenomenon in cancers [14]. Additionally, the virus contains a chimeric capsid which contains adenovirus serotype 3 knob with an adenovirus serotype 5 shaft and tail. Treatment doses ranged from 3 × 10^9^ to 4 × 10^12^ viral particles. Supplementary Table 1 provides a detailed overview of the number of patients per dose level in each trial. Treatment with TILT-123 continued with local virus administration seven days following the initial i.v. dose [23]. Combination therapy with anti-PD-1 (pembrolizumab) in PROTA and tumor infiltrating lymphocyte (TIL) therapy in TUNINTIL were initiated on day 36. However, the i.v. phase is the sole focus of this present work. Overall survival (OS) data was retrieved from electronic clinical data records and data collection cutoff was on 1 June 2024.

## Immunohistochemistry and multiplex immunofluorescence of tumor biopsies

**Fig. 1:**
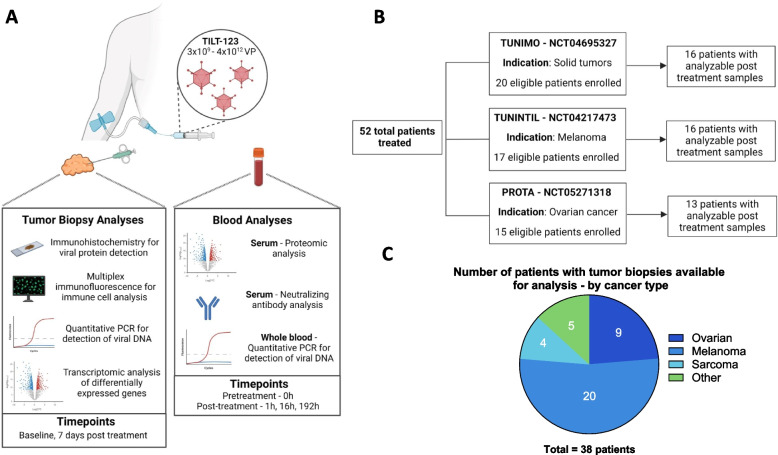
Overview of sample collection analysis across trials. **A** Sample analysis methods for tumor biopsies and blood as well as collection timepoints. **B** Summary of individual trials and number of patients with samples available for analysis. **C** Total number of analyzed patient tumor biopsies by cancer type

Tumors were biopsied when feasible using an 18-gauge biopsy needle at baseline and 7 days post-treatment (herein referred to as day 8 biopsy) (Fig. 1A). After collection, tumor biopsies were formalin fixed and paraffin embedded, sectioned, and stained. Sample quality was assessed using hematoxylin and eosin (H&E) stained samples, and those passing quality control were then used for immunohistochemistry (IHC) and multiplex immunofluorescence (mIF) analysis. IHC detection of viral proteins was performed using anti-adenovirus-hexon antibody (Millipore, AB1056, 1:1000) for PROTA samples and anti-adenovirus-5 E1A antibody (sc-58658, Santa Cruz Biotechnology, 1:900) in TUNIMO and TUNINTIL samples. Antibodies used for mIF staining are listed in Supplementary Table 2. Stained samples were scanned on Zeiss Azio Scan Z.1 (Carl Zeiss AG, Oberkcohen, Germany). Tumor delineation and immune cell quantification were performed using Cell profiler (4.2.5) or using Indica Labs HALO software (Oracle Bio).

## Viral DNA detection in whole blood and tumor biopsies

DNA was extracted from pre-treatment and post-treatment whole blood and tumors using the NucleoSpin®96 Tissue Kit (Macherey Nagel Bioanalysis, Germany) or MagMax DNA Multi-Sample Ultra 2.0 Kit (Thermo Fisher, MA, USA). Timepoints analyzed for whole blood included pre-treatment and post-treatment 1, 16, and 192 hours (h). Timepoints analyzed for tumor biopsies included pre-treatment, and post-treatment (day 8). The presence of viral DNA was detected by quantitative polymerase chain reaction (qPCR) by targeting the IRES-hIL2 region of TILT-123, which is uniquely expressed by the TILT-123 virus and not found in the human genome or any other therapies or vaccines for use in humans [14]. qPCR cycle threshold values were converted using an established standard curve into viral particles and values were reported as VP/mL or VP/ug.

## Serum proteomic analysis

Serum was separated from whole blood collected pre-treatment and post-treatment (16 h and 192 h) and used for proteomic analysis with the Olink Target 96 Immuno-Oncology Panel (Thermofisher, MA, USA) for the TUNIMO and TUNINTIL trials, and with the Olink Target 48 Cytokine Panel (Thermofisher, MA, USA) for PROTA patient analysis. Data reporting units were normalized protein expression (NPX) for TUNIMO and TUNINTIL and reported as absolute protein concentration in pg/ml for PROTA. Bridging samples were used to normalize protein levels across runs and the resulting data was analyzed in R Studio (4.3.1). Statistical differences between timepoints were analyzed using Mann–Whitney U tests, and proteins were considered to be differentially expressed when p-value < 0.05. A full list of all significantly upregulated serum proteins 192 h post-treatment in TUNIMO can be found in Supplementary Table 3.

## Neutralizing antibody analysis

Anti-adenovirus antibodies were analyzed using a previously described neutralizing antibody assay [24]. Briefly, serial dilutions of patient sera were added onto plated A549 cells followed by the addition of replication-incompetent luciferase-expressing Ad5/3-Luc1 virus. Luciferase expression was then detected using a commercial kit (Promega, WI, USA). The dilution which blocked 80% of luciferase expression was defined as the neutralizing antibody titer.

## Transcriptomic analysis of tumor biopsies

Fragments of tumor biopsies were snap frozen for RNA extraction and transcriptomic analysis with NanoString nCounter® gene expression analysis using the nCounter® Digital Analyzer (NanoString Technologies, WA, USA). Gene expression was analyzed using the nCounter® HumanPanCancer Immune Profiling Panel (catalog number XT-CSO-HIP1-12). Adenovirus gene expression was analyzed with the inclusion of additional adenovirus-specific genes (*hexon, fiber, and E1A*), specific target sequences can be found Supplementary Table 4. A full list of analyzed genes and overall gene expression can be found in the.xls Additional File 1. Differential gene expression between groups was tested using either a t-test or Mann–Whitney U test (for non-normally distributed data), and genes were considered differentially expressed if p-value < 0.05. Genes were functionally annotated using R package "ClusterProfiler", and gene ontology (GO) over-representation analysis was performed to identify enriched pathways [25]. Analysis was completed using R Studio (4.3.1), and pathways with p-value < 0.01 were considered significant.

## Statistical analyses and data presentation

Statistical analysis was performed using GraphPad Prism (10.1.2) or R Studio (4.3.1). Comparisons between groups were performed using Kruskal–Wallis tests, Mann–Whitney U-tests or two-tailed t-tests, as specified in the figure legends. Correlation analysis between variables was performed using Spearman’s rank correlation coefficient. For overall survival analyses, the Mantel-Cox Log-rank were performed, as specified in the figure legends. BioRender was used to created graphical illustrations (Fig. 1 A-B).

## Role of funding source

TILT Biotherapeutics was involved in designing the trials, data analysis, data interpretation, writing, and submission of the report for publication.

## Results

### Patients

TUNIMO, TUNINTIL, and PROTA are three separate phase I clinical trials targeting solid tumors, melanoma, and ovarian cancer respectively. The most common tumor types were melanoma (*n* = 20), ovarian cancer (*n* = 18), and soft-tissue sarcoma (*n* = 7). The median patient age was 61 years. Most participating patients had been heavily pretreated, having received a median of 4 previous systemic lines of therapy. Overall, 52 patients were treated with 3 × 10^9^ – 4 × 10^12^ VP of TILT-123 across these three trials, and of all treated patients, 45 patients had biological samples (tumors, whole blood, or serum) collected during the i.v. phase of each trial (Fig. 1A-B). 38 patients had post-treatment biopsies suitable for IHC analysis of viral proteins (Fig. 1C).

## Safety of i.v. TILT-123

Safety of a single i.v. injection of TILT-123 was assessed from the initial TILT-123 administration on day 1 to the start of i.t. virus administrations beginning on day 8. While i.t. injections were administered starting on day 8 as part of the trial protocol, this analysis focuses on the safety and immunomodulatory effects after the initial i.v. administration of TILT-123 on day 1. The dose was escalated from 3 × 10^9^ to 4 × 10^12^ VP in a standard 3 + 3 design. The most common reported adverse events were fever (*n* = 11, 21.2%), decreased lymphocyte count (*n* = 6, 11.5%) and nausea (*n* = 5, 9.6%) (Table 1). Three patients experienced a grade 4 lymphocyte count decrease, and one patient experienced a grade 3 decrease. Among these patients, one received 1 × 10^12^ VP, another received 2 × 10^12^ VP, and two patients received 4 × 10^12^ VP of TILT-123. These events did not cause symptoms, were transient, and did not result in early trial termination, with patients recovering without any treatment. Additionally, one patient receiving 3 × 10^9^ VP experienced a grade 3 fever and recovered. Only one patient experienced a grade 1 infusion site reaction. Overall, the i.v. administration of TILT-123 was well tolerated with only five patients experiencing grade 3 or greater events related to treatment without dose limiting toxicity.

**Table 1:**
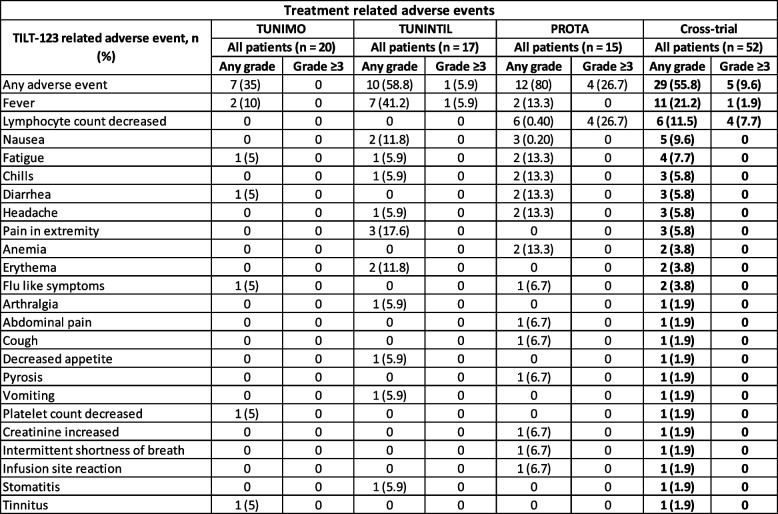
Adverse events related to single i.v. administration of TILT-123. Adverse events were judged and reported by trial investigators. Reported as number of patients experiencing an event of any grade and grade ≥3, per individual trial and cross-trial

No signs of liver damage were detected after single i.v. TILT-123 administration, as measured by liver enzymes alanine aminotransferase (ALT), aspartate aminotransferase (AST), and lactate dehydrogenase (LDH) levels (Supplementary Fig. 1A). Additionally, there was no correlation between dose of virus received and post-treatment liver enzyme levels (Supplementary Fig. 1B).

## Virus bioavailability

**Fig. 2:**
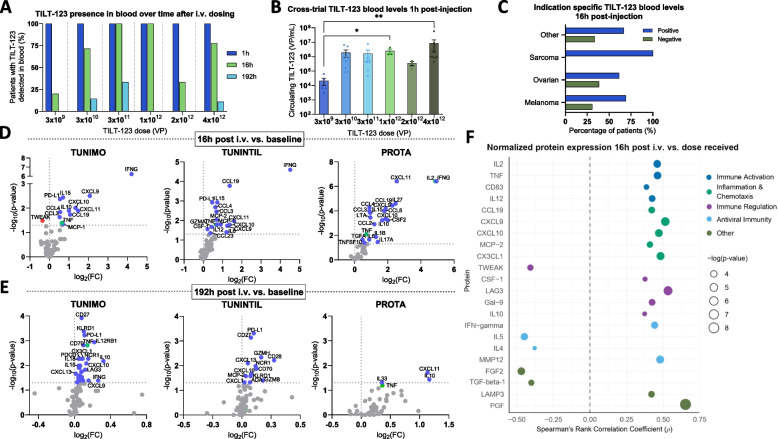
Detection of TILT-123 in blood and serum protein changes post-treatment. **A** Percentage of patients with detectable levels of TILT-123 detected by quantitative PCR (qPCR) in whole blood 1 h, 16 h, and 192 h post i.v. TILT-123 administration grouped by dose received. **B** Cross-trial results of circulating TILT-123 levels in blood 1-h post-injection, grouped by dose received. Data presented as mean ± SEM. Comparisons were evaluated using Kruskal–Wallis test with Dunn’s multiple comparisons test where *p < 0.05 and **p < 0.01. **C** Percentage of patients with detectable levels of TILT-123 in blood 16 h post-injection by cancer type. Volcano plots illustrating changes in serum proteins 16 **D** and 192 h **E** post TILT-123 administration compared to baseline, per individual trial TUNIMO, TUNINTIL and PROTA. Grey dots indicate genes which do not pass threshold values of *p* < 0.05. Statistical differences between groups were assessed using Mann–Whitney U tests, where non-significant results were p > 0.05. A full list of differentially expressed proteins 192 h post-injection (TUNIMO) can be found in Supplementary Fig. 1B. **F** Spearman correlation analysis of normalized protein expression values and TILT-123 dose (TUNIMO & TUNINTIL). Only significant results are displayed (*p* < 0.05)

A cross-trial analysis of TILT-123 in whole blood 1 h post i.v. administration demonstrated that all patients had detectable levels of viral DNA, regardless of the dose received (Fig. 2A). 16 h post-administration, detection of TILT-123 was more variable: only 20% of patients had detectable TILT-123 DNA at the lowest dose. In contrast, at doses of 3 × 10^11^ and 1 × 10^12^ VP, all patients had detectable TILT-123 in blood at the same timepoint (Fig. 2A). Interestingly, a minority of patients (*n* = 4) had detectable virus in blood 192 h post-administration; these patients had received 3 × 10^10^, 3 × 10^11^ or 4 × 10^12^ VP (Fig. 2A). Cross-trial analysis of TILT-123 levels in blood 1-h post-injection found a statistically significant increase in the amount of circulating TILT-123 DNA between patients receiving 3 × 10^9^ VP and 1 × 10^12^ VP (p = 0.0158), as well as between patients receiving 3 × 10^9^ VP and 4 × 10^12^ VP (p = 0.0020, Fig. 2B). When stratifying the patients by their cancer type, at the 16-h post-administration timepoint all sarcoma patients (*n* = 4) had detectable levels of virus, in comparison to melanoma (69.23%, *n* = 9), ovarian (61.54%, *n* = 8), and other cancer types (66.67%, *n* = 2) (Fig. 2C).

## Systemic response and immunogenicity

Serum isolated from whole blood pre-treatment and post-treatment (16 h and 192 h) was analyzed to determine systemic response to i.v. TILT-123. Across all trials, a significant increase in pro-inflammatory mediators such as IFNG, TNF, CXCL9, CXCL10, CXCL11, CCL3, and CCL4 (Fig. 2D) was noted 16 h after i.v. administration of TILT-123 when compared to baseline values. Additionally, PD-L1 was found to be significantly upregulated at the same timepoint in TUNIMO and TUNINTIL patients. Inflammatory chemokines CCL8, CXCL10, CXCL13 were found to be upregulated in serum 192 h (seven days) post-treatment in TUNIMO and TUNINTIL patients (Fig. 2E). Furthermore, an upregulation of markers related to adaptive immunity (NCR1, CD70, CD83, CD5) and immune system regulation (IL10, PD-L1, KLRD1) was noted. At the same timepoint, PROTA patients had significantly upregulated pro-inflammatory chemokines CXCL11 and IL33 as well as anti-inflammatory cytokine IL-10 (Fig. 2E).

Levels of IL2 and TNFα, cytokines expressed by immune cells and encoded by TILT-123, positively correlated with dose increase (Fig. 2F). Moreover, a statistically significant positive correlation (*p* < 0.05) between proteins involved in immune activation (CD83, IL12), inflammation chemotaxis (CCL19, CXCL9, CXCL10, MCP-2, CXCL1), immune regulation (TWEAK, CSF-1, LAG3, Gal-9, IL10) and antiviral immunity (IFN-gamma, IL5, IL4, MMP12) was noted in a correlative analysis of normalized protein expression values 16 h post-treatment and the dose of TILT-123 received (Fig. 2F). Additionally, a moderate positive correlation between the dose of TILT-123 and the amount of circulating virus 1 h post i.v. administration was observed (p = 0.0003), while no correlation was observed at the 16 h timepoint (Supplementary Fig. 2A).

Overall, analysis of baseline serum samples revealed that half of patients (*n* = 23) had detectable levels of neutralizing antibodies against TILT-123 (defined as titer less than 1:64), while the other half had undetectable levels (titer less than 1:64), (Supplementary Fig. 2B). Analysis of serum collected on day 8 showed that all but one patient developed neutralizing antibodies against TILT-123, regardless of the administered virus dose (Supplementary Fig. 2C). Additionally, in patients with positive detection of TILT-123 in post-treatment biopsies by qPCR or IHC, high neutralizing antibody titers (1:4096 and 1:16,384) were observed (Supplementary Fig. 2D). Patients with TILT-123 negative biopsies showed a wide range of antibody titers, including both high and lower levels (Supplementary Fig. 2E).

## TILT-123 transduction of tumors

**Fig. 3:**
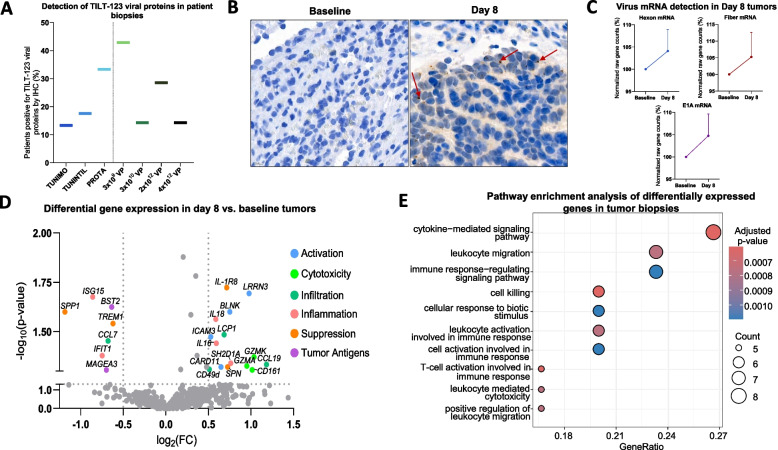
Detection of viral proteins and transcriptomic changes in biopsies. **A** Percentage of patients positive for viral proteins (E1A or hexon) in day 8 biopsies detected by IHC, per trial and by dose received. **B** IHC image of day 8 spleen biopsy from an epithelial ovarian cancer patient 30210. Red arrows highlighting speckled brown diaminobenzidine (DAB) stain indicating the presence of hexon. **C** Normalized gene counts of viral mRNA (*hexon, fiber, and E1A*) in baseline and day 8 biopsies detected using Nanostring nCounter® gene expression analysis. Nanostring analysis was performed on 14 tumor biopsies, including 9 biopsies from patients in the TUNIMO trial and 5 biopsies from patients in the PROTA trial. Data presented as mean ± SEM. **D** Volcano plot of differentially expressed genes analyzed by Nanostring nCounter® in day 8 biopsies compared to baseline biopsies, grouped by immunological function. Grey dots indicate genes which do not pass threshold values of –log_10_(*p*-value) > 1.3 and log_2_(FC) > 0.5 or < -0.5. Statistical differences between groups were assessed using either t-tests or Mann–Whitney U tests, where non-significant results were *p* > 0.05. **E** Pathway enrichment analysis of differentially expressed genes (DEGs) in day 8 biopsies

Tumor biopsies were assessed to evaluate the ability of TILT-123 to transduce tumors through systemic delivery. Immunohistochemistry analysis of available biopsies (*n* = 38) for the presence of adenoviral proteins (E1A or hexon) noted positivity in 13.3% of TUNIMO patients (*n* = 2 out of 15), 17.6% in TUNINTIL patients (*n* = 3 out of 17), and 33.33% in PROTA patients (*n* = 2 out of 6) (Fig. 3A). Additionally, one out of seven post-treatment tumor biopsies analyzed in TUNIMO was positive for TILT-123 DNA by qPCR. These positive tumor biopsies came from patients treated with a wide dose range of TILT-123 (3 × 10^9^ – 4 × 10^12^ VP), indicating the ability of the virus to transduce tumors across varying dose levels (Fig. 3A). An example of positive hexon staining is demonstrated in an ovarian cancer patient treated with 4 × 10^12^ VP (Fig. 3B). Interestingly, transcriptomic analysis of tumor biopsies was able to capture TILT-123 transcription in tumor cells through the expression of *hexon*, *fiber* and *E1A* adenovirus genes seven days post-TILT-123 i.v. administration (Fig. 3C).

In addition to virus detection in tumors, a comprehensive transcriptomic analysis of genes associated with immune response was performed in tumor biopsies. In patients from TUNIMO and PROTA, tumors presented a statistically significant upregulation of genes involved in immune activation (*LRRN3, BLNK, ICAM3, CARD11*), cytotoxicity (*GZMA, GZMK, CD161*), infiltration (*LCP1, CCL10, CD49d*), inflammation (*IL16, IL18, SH2D1A*), and suppression (*IL-1R8, SPN*) when compared to baseline tumors (Fig. 3D). Day 8 biopsies also demonstrated a significant downregulation of genes involved in inflammation (*IFIT1, ISG15*), immune suppression (*TREM1, SPP1*), and infiltration (*CCL7*) (Fig. 3D). Additionally, there was a significant downregulation of tumor antigen expression (*BST2, MAGEA3*) in post-treatment biopsies (Fig. 3D). Altogether, these findings suggest that TILT-123 is able to transduce tumors via the i.v. route, and stimulate genes associated with cell killing and adaptive immunity formation. Pathway enrichment analysis of differentially expressed genes further supports these findings as many of the genes were statistically associated with immune-related pathways and cell killing (Fig. 3E).

**Fig. 4:**
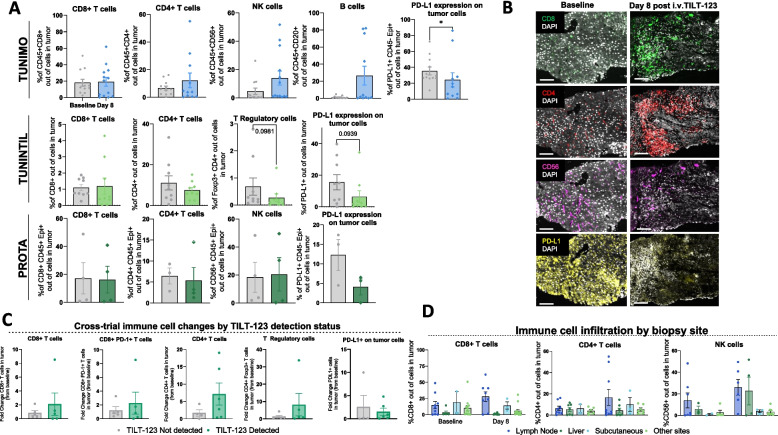
Immune cell marker expression in tumor biopsies. **A** Percentage of cells in baseline and day 8 tumors expressing various immune cell markers, per trial. TUNIMO – CD8 + , CD4 + , CD56 + , CD20 + and PD-L1 + expressing cells in the tumor. TUNINTIL – CD8 + , CD4 + , Foxp3 + , and PD-L1 + expressing cells in the tumor. PROTA – CD8 + , CD4 + , CD56 + , and PD-L1 + expressing cells in the tumor. Data presented as mean ± SEM. Differences between groups were compared using Mann–Whitney U tests where *p < 0.05. **B** Multiplex immunofluorescence of baseline and day 8 liver biopsies from epithelial ovarian cancer patient 30103 indicating changes in CD8 + , CD4 + , CD56 + , and PD-L1 + expressing cells after TILT-123 treatment. **C** Cross-trial comparison of changes in CD8 + , CD8 + PD-1 + , CD4 + , Foxp3 + , PD-L1 + expressing cells grouped by detection of adenoviral proteins in tumors by IHC. Data presented as mean ± SEM. **D** CD8 + , CD4 + T cell and CD56 + expressing cells in tumor cells of post-treatment biopsies, grouped by biopsy site. Data presented as mean ± SEM

Multiplex immunofluorescence showed changes in different immune cell populations infiltrating tumors following TILT-123 i.v. administration. An increase in lymphocyte populations including CD4 + T, CD8 + T, and CD56 + cells was observed to different extents in tumors from TUNIMO after TILT-123 treatment, although not statistically significant (Fig. 4A). In PROTA and TUNINTIL, quantification results for the above-mentioned lymphocyte populations fluctuated between baseline and post TILT-123, but they remained comparable (Fig. 4A). Interestingly, across all trials, there was an observable trend in decreased PD-L1 expression on tumor cells, with significant differences in TUNIMO (*p* = 0.0439) (Fig. 4A). Downregulation of anti-inflammatory response was also observed in regulatory T (Treg) cells post-treatment (*p* = 0.0981) in TUNINTIL. Figure 4B illustrates mIF of a liver biopsy from an epithelial ovarian cancer patient, where there is an observable increase in tumor infiltrating CD8 + and CD4 + T cells, and a decrease in CD56 + and PD-L1 cells after TILT-123 treatment.

Cross-trial analysis of patients divided by tumor biopsy positivity for TILT-123 by IHC, demonstrated a clear trend in increased fold change from baseline in CD8 + , CD8 + PD-1 + , CD4 + and CD4 + Foxp3 + T cells in biopsies with confirmed TILT-123 transduction, as well as a decreased fold change in PD-L1 + cells, indicating an immunological response associated with TILT-123 transduction (Fig. 4C). When tumor biopsies were stratified by their biopsy sites, the most common sites with increased immune cell infiltration were lymph node biopsies, liver, and subcutaneous biopsies (Fig. 4D).

## Analysis of TILT-123 tumor transduction and patient outcomes

**Fig. 5:**
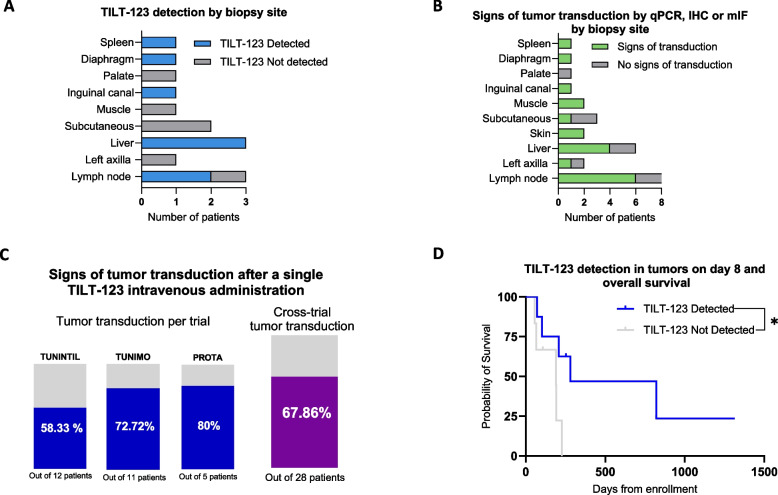
Analysis of TILT-123 detection and tumor transduction in post-treatment biopsies. **A** Number of patients per biopsy site grouped by presence of TILT-123 in day 8 biopsies as assessed by qPCR or IHC. Only patients with results available for both assays were included. **B** Number of patients per biopsy site grouped by signs of tumor transduction. Signs of tumor transduction were assessed using tumor qPCR, IHC or mIF results and only patients with results available for at least two of the assays were included. **C** Percentage of patients showing signs of tumor transduction per individual trial and cross-trial. **D** Association of OS and positive TILT-123 detection (*n* = 8) or negative TILT-123 detection (*n* = 6) in day 8 biopsies as assessed by qPCR or IHC. Groups were compared using log-rank (Mantel-Cox) test where *p < 0.05

To assess the possible trend in tumor sites and successful TILT-123 transduction, patients were categorized by biopsy site and status of TILT-123 detection, or signs of tumor transduction (defined as increase in immune cells). The most common biopsy sites with detectable TILT-123 were the liver (*n* = 3), and lymph node biopsies (*n* = 2) followed by spleen, diaphragm (right crus), and inguinal canal biopsies (*n* = 1 each) (Fig. 5A). Sites negative for TILT-123 detection included the palate (*n* = 1), muscle (*n* = 1), subcutaneous tissue (*n* = 2), left axilla (*n* = 1), and one lymph node biopsy. To further assess the effect of tumor biopsy location on tumor transduction, patients were grouped based on signs of tumor transduction by presence of TILT-123 or enhanced immune cell infiltration by mIF. The results indicated that 75% of lymph node (*n* = 6), 66.67% of liver (*n* = 4), as well as 100% of skin and muscle biopsies (*n* = 2 each) were most commonly positive for signs of tumor transduction (Fig. 5B).

Overall, cross-trial analysis of patients enrolled in all three studied trials demonstrated that 67.86% of patients (*n* = 20 out of 28) had signs of tumor transduction after a single TILT-123 i.v. dose (Fig. 5C). Although 38 biopsies were collected, only 28 were included in this analysis as they had results for at least two out of three detection methods (qPCR, IHC, or mIF). Survival analysis of patients with TILT-123 detected in tumors demonstrated statistically significantly increased overall survival, with a median survival of 280 days from enrollment compared to 190 days for patients where TILT-123 could not be detected (*p* = 0.0405) as of 1 June 2024 (Fig. 5D).

## Discussion

Herein, we report the findings of three individual phase I dose escalation clinical trials with a focus on the single i.v. administration of TILT-123 employed during the first week in each of these trials. Our findings demonstrated that systemic delivery of TILT-123 was safe, able to transduce tumors, and induce immunomodulatory effects, highlighting the viability of the systemic delivery approach. The potential of i.v. delivery of OVs is further supported by a previous trial of VCN-01, an oncolytic adenovirus encoding for hyaluronidase (VCN-01), administered alone or in combination with gemcitabine and nab-paclitaxel in patients with advanced solid tumors (NCT02045602) [26]. The recent FDA grant of orphan drug designation to VCN-01 for the treatment of pancreatic cancer in 2023 further emphasizes the promise of i.v. delivery of OVs.

Virus bioavailability analysis is essential to elucidate potential mechanisms of OV action and to meet safety regulatory requirements. Out of the timepoints analyzed, whole blood collected 1 h post-treatment had the highest amount of TILT-123 DNA detected. Notably, all sarcoma patients had detectable levels of TILT-123 in whole blood at the 16 h timepoint, compared to the other tumor types which ranged from 62- 69% positivity. Given that viral replication, which typically takes around 48 h, is unlikely at this early timepoint, the detection of virus in the blood at this time could be caused by impaired entry of the virus into tumor cells by a dense extra cellular matrix (ECM) [27]. Although generally understudied, sarcomas are thought to produce large amounts of ECM components due to their mesenchymal differentiation [27]. ECM components such as hyaluronan have been shown to impair the ability of OVs to enter tumor cells, and this could cause the virus to remain in circulation for a longer period of time [28, 29]. Interestingly, a minority of patients (*n* = 4) had detectable levels of viral DNA 192 h post-injection. The tumor types of these patients were melanoma (*n* = 3) and ovarian cancer (*n* = 1). Detectable levels of viral DNA at this timepoint could be attributed to ongoing viral replication in tumors and dissemination into the circulatory system. Among these patients, two are alive at data cutoff, while the other two patients had OS times of 257 and 557 days, compared to a median OS of 191 days in patients with undetectable TILT-123 in blood at the same timepoint.

Analysis of serum proteins found a robust pro-inflammatory response shortly (16 h) after i.v. administration of TILT-123, followed by a more regulated adaptive immune response 192 h (seven days) post-treatment. The initial increase in pro-inflammatory cytokines and chemokines indicated an acute activation of the immune system, likely driven by the initial recognition of the adenovirus. Traditionally, antiviral immunity is thought to be detrimental to OV therapy efficacy by restricting viral replication and dissemination. However, the antiviral immune response may paradoxically enhance antitumor effects. Antiviral immunological events occurring within the TME can induce a strong pro-inflammatory response, resulting in the recruitment and activation of various immune cells (natural killer cells, dendritic cells, and T cells), thus augmenting the antitumor immune response [30, 31]. Interestingly, we found that the presence of adenoviral NAbs did not hinder the ability of TILT-123 to transduce tumors, even at the highest detectable titers. NAb levels were assessed using a luciferase-based assay, which specifically measures the neutralizing capacity of anti-adenoviral antibodies, unlike ELISA-based assays that quantify the amount of antibodies. Similar findings, where NAbs did not interfere with OV treatment, have been made in other clinical trials investigating OVs, such as VCN-01 and oncolytic herpes virus (CAN-3110). VCN-01 therapy responders were found to have a higher fold change in post-treatment NAbs compared to non-responders [26]. Additionally, a correlation was observed between glioblastoma patients with pre-existing herpes simplex virus 1 NAbs and increased survival following CAN-3110 treatment [32]. It is hypothesized that this phenomenon could be explained by NAbs serving as an indicator of a "healthier" immune system more likely to respond to immunotherapy, or destruction of virus-infected cells through antibody-dependent cell-mediated cytotoxicity [32, 33]. Studies have also found that pre-existing virus specific CD8 + memory T cells populate tumors, and activation of these cells could contribute to tumor clearance, although these cells were not analyzed in the clinical study presented here [34, 35]. Our focus was on evaluating broader immune cell populations and the direct impact of TILT-123 on overall immune infiltration. Future studies could benefit from including a more detailed analysis on antiviral immunity.

The ability of TILT-123 to transduce tumors through systemic delivery was demonstrated by changes in gene expression, presence of viral proteins, and enhanced immune cell infiltration in post-treatment biopsies. Transcriptomic analysis of tumors found an upregulation of genes related to cytokine-mediated signaling, leukocyte migration, and cell killing, indicating an immune response to TILT-123. Post-treatment tumors also exhibited a significant downregulation of tumor antigens (*MAGEA3, BST2*) when compared to baseline. This could be explained by antigen escape, a phenomenon where tumor cells lose or reduce expression of specific antigens in response to killing of antigen-expressing cancer cells by tumor antigen-specific CD8 + T cells, which is a known occurrence in patients treated with T-cell immunotherapies, such as CAR T-cell therapy [36, 37]. However, it is important to note that the results come from a heterogeneous population of tumor types, including ovarian, melanoma, non-small cell lung cancer, myxoid liposarcoma, neuroendocrine carcinoma of the bladder, and mucinous carcinoma of the appendix. The expression of *MAGEA3* and *BST2 *may vary significantly across these different malignancies. Cross-trial detection of viral proteins, hexon or E1A, in post-treatment biopsies was low, at 18.42% positivity. This low detection rate could be due to several factors. First, the subdivision of tumor biopsies into smaller fragments for separate assays, potentially resulting in the analysis of uninfected tissue fragments. Second, the sensitivity of the assay and biopsy timing may have influenced protein detection limits. Detection of E1A and hexon are expected to have low sensitivity, as *E1A* is the first gene to be expressed in the viral replication cycle 1–2 h post-infection, while *L3* (which encodes the hexon protein) is expressed in the later stages (36–48 h) after infection, just before the cells are lysed [38, 39]. Thus, the window of detection for both viral proteins is short. In the analysis of PROTA tumor biopsies, 2 out of 6 patients showed detection of hexon, a higher positivity rate than in other trials, likely due to improved detection methods developed over time, although caution should be used in interpreting low numbers. Interestingly, five of the patients with positive biopsies for E1A or hexon had very high levels of NAbs (titers of 1:4096 or 1:16,384), suggesting that NAbs did not impede TILT-123 from reaching tumor sites. Only one tumor biopsy was positive for TILT-123 DNA, likely due to the low sensitivity of the qPCR assay. Since TILT-123-infected tumor cells die shortly after viral genome amplification, there is a rather short window for genome detection in biopsies, which are collected on day 8.

We found enhanced immune cell infiltration of post-treatment biopsies with confirmed TILT-123 infection by IHC. This might be the most sensitive of the detection methods used in this study, as it assesses the results of virus replication that had already occurred. In contrast, direct viral detection methods rely on the presence of TILT-123 at the exact time and location of the biopsy, make them more temporally constrained. Interestingly, a post-treatment liver biopsy illustrated in Fig. 4B demonstrated a co-localization of CD8 + T cells and CD56 + NK cells. This may suggest a potential intercellular communication that plays a role in tumor control. A recent study investigating spatial co-localization in non-small cell lung cancer demonstrated a positive correlation between CD8 + and CD56 + cell counts indicating coordinated infiltration and functional interaction between these immune cell populations [40]. Moreover, spatial analysis in the same study found that NK and CD8 + T cells were present in clusters marked by IFN-gamma activity, suggesting enhanced immune activity in those regions. The observed co-localization of these two cell types in this tumor biopsy may reflect their concurrent targeting of tumor cells within these clusters. Notably, lymph node and liver tumor biopsies consistently exhibited enhanced immune cell infiltration and coincided with being most commonly positive for detection of TILT-123, compared to other biopsy sites. Additionally, a subcutaneous biopsy from a nodular melanoma patient in TUNIMO demonstrated a notable increase in immune cell infiltration compared to other biopsy sites. Immune cell infiltration has been reported to serve as a favorable prognostic indicator for cancer immunotherapy response in melanoma [41, 42]. This patient experienced an OS time of 377 days, exceeding the median OS of 109 days seen in other melanoma patients enrolled in the same trial. The enhanced immune cell infiltration of lymph node metastasis biopsies compared to other sites is expected, given their role as essential organs of the adaptive immune system and major sites of B and T cells [43]. Predominant detection of viral proteins in the lymph nodes may be attributed to the migration of antigen-presenting cells loaded with viral antigens to the lymph nodes for presentation to T cells, or due to the virus reaching metastatic lymph nodes via the lymphatic circulation [44–46]. The consistent detection of TILT-123 in all liver biopsies could be attributed to the interplay between virus clearance mechanisms and the liver’s unique immune microenvironment. As with any drug administered systemically, viral particles accumulate in the liver due to its dual blood supply by the portal vein and the hepatic artery, as evidenced by virus uptake by hepatic macrophages (Kupffer cells) [47, 48]. Additionally, immune tolerance mechanisms of the liver, which prevent immune activation against innocuous antigens, can allow viruses to chronically persist in the liver, as seen with hepatitis B and hepatitis C viruses [49, 50]. Kupffer cells may not be able to clear all viral particles and the inhibitory immune microenvironment of the liver may facilitate the replication of TILT-123 in liver metastases allowing for their detection in biopsies. However, it is important to consider sampling bias in these findings which are based on a limited number of biopsies primarily taken from relatively accessible tissues, such as lymph nodes. It is possible that TILT-123 also localizes to other, less accessible tissues that have not been biopsied in these trials.

While survival analysis demonstrated a statistically significant increase in OS in patients with detectable TILT-123 in post-treatment biopsies, it is also important to note that patients received subsequent treatments including i.t. TILT-123, TIL therapy, or pembrolizumab. As a result, the observed OS benefit may reflect the cumulative effect these therapies. Nonetheless, the survival benefit associated with early detection of TILT-123 is an intriguing finding which may serve as a potential biomarker of treatment efficacy.

## Conclusion

This study found that a single i.v. administration of TILT-123 was safe, and able to transduce tumors in patients who had failed multiple lines of treatment. Transduction of tumors was evidenced by the presence of viral proteins, upregulated immune responses observed through gene expression profiling, and enhanced immune cell infiltration confirmed by immunofluorescence. Most importantly, the detection of TILT-123 in day 8 tumors correlated with improved OS, emphasizing the potential benefit of successful TILT-123 transduction of tumors. While future clinical trials should incorporate further mechanistic studies to better understand the mechanism of TILT-123, these early findings indicate that the virus is functioning as expected based on its preclinical profile. Although this study focused on a single i.v. injection of TILT-123, virus delivery and subsequent treatment efficacy could possibly be enhanced by performing multiple injections. Regimens featuring multiple i.v. injections of TILT-123 are now being studied (Cohort 7 in NCT04695327, NCT06125197). The results of this study highlight the potential of TILT-123 as a promising i.v. therapy for metastatic cancers.

## Supplementary Information

Supplementary Material 1.

Supplementary Material 2:  Supplementary Table 1. Number of patients per dose received grouped by trial and cross-trial.

Supplementary Material 3:  Supplementary Table 2. List of antibodies and dilutions used for multiplex immunofluorescence. *Exclusively used in TUNINTIL sample analysis.

Supplementary Material 4:  Supplementary Table 3. List of all differentially expressed proteins at 192h post-injection in TUNIMO patients.

Supplementary Material 5:  Supplementary Table 4. Target mRNA sequences of adenoviral genes used in Nanostring analysis.

Supplementary Material 6:  Supplementary Fig. 1. Liver enzyme changes and correlation with treatment dose. A. Changes in liver enzymes alanine aminotransferase (ALT), lactate dehydrogenase (LDH), and aspartate transaminase (AST). Data are presented as mean ± SEM. Differences between timepoints were compared using Mann–Whitney U test where ns > 0.05. LLN = lower limit of normal. ULN = upper limit of normal. B. Spearman correlation of dose received and day 1 post-treatment liver enzyme levels.

Supplementary Material 7:  Supplementary Fig. 2. Neutralizing antibody levels across trials. A. Spearman correlation analysis of circulating levels of TILT-123 1 h or 16 h post-injection and dose received where ***p < 0.001. B. Number of patients with NAbs detected or not detected at baseline. C. Cross-trial baseline and post-treatment neutralizing antibody titers, by dose received. Data are presented as mean ± SEM. D. NAb titers of patients with TILT-123 detected in day 8 biopsies. Patients 20202, 20204, and 20205 were enrolled in TUNIMO. Patients 10102, 10103 and 30209, 30210 were enrolled in TUNINTIL and PROTA trials, respectively. E. Neutralizing antibody titers in patients with TILT-123 negative day 8 biopsies. Patients 20203, 20101, 20206, 20219 were enrolled in TUNINTIL. Patients 10116 and 30207 were enrolled in TUNIMO and PROTA, respectively.

## Data Availability

Data and material are available upon reasonable request from TILT Biotherapeutics Oy.

## Abbreviations

i.t: Intratumoral
i.v: Intravenous
VP: Viral particles
TME: Tumor microenvironment
OVs: Oncolytic viruses
PAMPs: Pathogen-associated molecular patterns
DAMPs: Danger-assoociated molecular patterns
IL: Interleukin
TNFα: Tumor necrosis factor alpha
FDA: Food and Drug Administration
EMA: European Medicines Agency
NAbs: Neutralizing antibodies
VP: Viral particles
TIL: Tumor infiltrating lymphocyte
OS: Overall survival
H&E: Hematoxylin and eosin
IHC: Immunohistochemistry
mIF: Multiplex immunofluorescence
qPCR: Quantitative polymerase chain reaction
NPX: Normalized protein expression
ALT: Aminotransferase
AST: Aspartate aminotransferase
LDH: Lactate dehydrogenase
GO: Gene ontology
Treg: Regulatory T cell
