# Supplementary material for: Single intravenous administration of oncolytic adenovirus TILT-123 results in systemic tumor transduction and immune response in patients with advanced solid tumors
Source: J Exp Clin Cancer Res. 2024 Nov 6;43:297. doi: 10.1186/s13046-024-03219-0 (PMC11539705; doi:10.1186/s13046-024-03219-0)
Supplement: Supplementary file 2 — Supplementary Material 2: Supplementary Table 1. Number of patients per dose received grouped by trial and cross-trial. [file 13046_2024_3219_MOESM2_ESM.pdf]

Supplementary Table 1

	TUNIMO	TUNINTIL	PROTA	Cross-trial
**Dose received (VP)**	**Number of patients**			
$3x10^{9}$	2	3	N/A	5
$3x10^{10}$	4	3	N/A	7
$3x10^{11}$	3	3	3	9
$1x10^{12}$	3	3	3	9
$2x10^{12}$	3	5	3	11
$4x10^{12}$	5	N/A	6	11
