# Supplementary material for: Single intravenous administration of oncolytic adenovirus TILT-123 results in systemic tumor transduction and immune response in patients with advanced solid tumors
Source: J Exp Clin Cancer Res. 2024 Nov 6;43:297. doi: 10.1186/s13046-024-03219-0 (PMC11539705; doi:10.1186/s13046-024-03219-0)
Supplement: Supplementary file 3 — Supplementary Material 3: Supplementary Table 2. List of antibodies and dilutions used for multiplex immunofluorescence. *Exclusively used in TUNINTIL sample analysis. [file 13046_2024_3219_MOESM3_ESM.pdf]

Supplementary Table 2

Antibody	Dilution	Catalog	Manufacturer
CD45	1:100	M0701	Dako
CD4	1:400<br>1:500*	ab133616	Abcam
CD8	1:300<br>1:500*	M7103<br>ab101500	Dako<br>Abcam
CD56	1:100	156R-94	CM
CD20	1:200	MS-340	Thermo
Foxp3	1:100<br>1:300*	ab20034<br>MAB8214	Abcam<br>R&D Systems
PD-1	1:150<br>1:200	LSB12784<br>ab137132	LSBio<br>Abcam
PD-L1	1:200<br>1:150*	13684	CST<br>CST
E cadherin	1:200	3195	CST
Pan cytokeratin	1:200	ab9377	Abcam
