# Supplementary material for: Single intravenous administration of oncolytic adenovirus TILT-123 results in systemic tumor transduction and immune response in patients with advanced solid tumors
Source: J Exp Clin Cancer Res. 2024 Nov 6;43:297. doi: 10.1186/s13046-024-03219-0 (PMC11539705; doi:10.1186/s13046-024-03219-0)
Supplement: Supplementary file 4 — Supplementary Material 4: Supplementary Table 3. List of all differentially expressed proteins at 192h post-injection in TUNIMO patients. [file 13046_2024_3219_MOESM4_ESM.pdf]

Supplementary Table 3

List of all differentially expressed proteins at 192h post-injection – TUNIMO					
Gene	$log_2(FC)$	$-log_{10}$(p-value)	Gene	$log_2(FC)$	$-log_{10}$(p-value)
Gal-9	0.029635	1.319550283	CXCL13	0.048801	1.904789772
EGF	0.062898	1.384473243	MIC-A/B	0.050129	1.989110658
CXCL9	0.156572	1.384473243	IL15	0.06269	1.989110658
CD83	0.096483	1.384473243	CXCL10	0.132718	2.077699372
IFNG	0.26407	1.384473243	IL10	0.322664	2.173027254
CD5	0.045107	1.451741376	IL18	0.047509	2.269937267
CD8A	0.043327	1.52069299	CX3CL1	0.090219	2.269937267
CCL4	0.072732	1.52069299	NCR1	0.153472	2.269937267
IL6	0.083213	1.52069299	PDCD1	0.062836	2.269937267
TNFRSF9	0.059148	1.592205916	TNF	0.143442	2.816479931
CSF-1	0.014013	1.666645234	CD70	0.098544	2.816479931
CRTAM	0.079346	1.743128228	IL12RB1	0.220898	2.935666338
FASLG	0.07646	1.743128228	PD-L1	0.111131	3.214419939
LAG3	0.118553	1.821722986	KLRD1	0.105133	3.369321899
MCP-2	0.05496	1.904789772	CD27	0.078939	3.913389944
