# Supplementary material for: Single intravenous administration of oncolytic adenovirus TILT-123 results in systemic tumor transduction and immune response in patients with advanced solid tumors
Source: J Exp Clin Cancer Res. 2024 Nov 6;43:297. doi: 10.1186/s13046-024-03219-0 (PMC11539705; doi:10.1186/s13046-024-03219-0)
Supplement: Supplementary file 5 — Supplementary Material 5: Supplementary Table 4. Target mRNA sequences of adenoviral genes used in Nanostring analysis. [file 13046_2024_3219_MOESM5_ESM.pdf]

Supplementary Table 4

Target mRNA	Sequence
Adenovirus 3 fiber knob	AATGCTACTACAAAGCAAGCGATGGTGCCCTTTTTCCGTTGGAAGTTACTGTTATGCTTAATAAACGCCTGCCAGATAGTCGCACATCCTATGTTATGAC
Adenovirus 5 hexon	ATGCAATTTTTCTCAACTACTGAGGCAGCCGCAGTCAATGGTGATAACTTGACTCCAAAAGTGGTATTGTATAGCGAAGACGTGGATATAGAAACTCCAG
Adenovirus 5 E1A	CGAGCAGCCGGAGCAGAGAGCCTTGGGTCCGGTTTCTATGCCAAACCTTGTACCGGAGGTGATCGATCTTACCTGCCACGAGGCTGGCTTTCCACCCAGT
