# Supplementary figures and images for: Single intravenous administration of oncolytic adenovirus TILT-123 results in systemic tumor transduction and immune response in patients with advanced solid tumors
Source: J Exp Clin Cancer Res. 2024 Nov 6;43:297. doi: 10.1186/s13046-024-03219-0 (PMC11539705; doi:10.1186/s13046-024-03219-0)

# Supplementary Figure 1

**A**

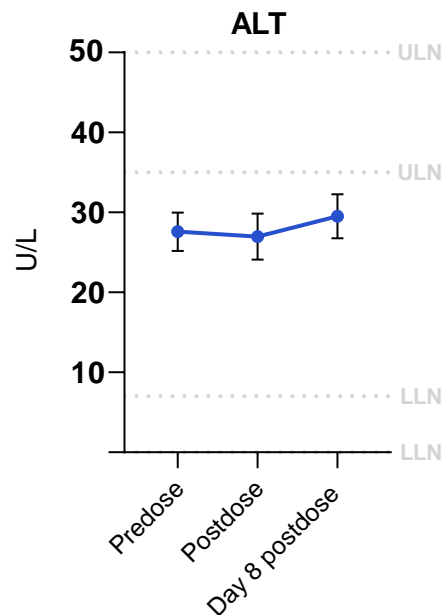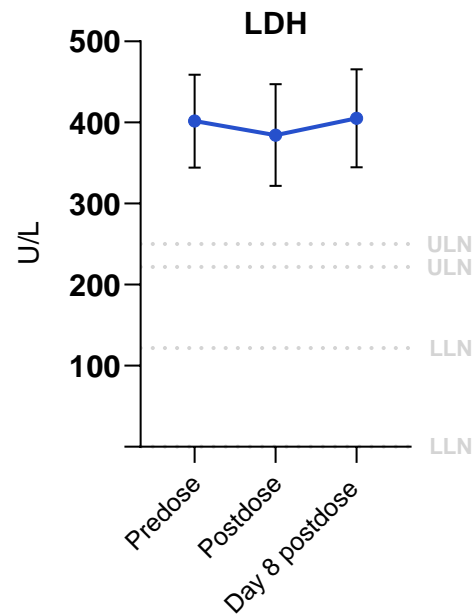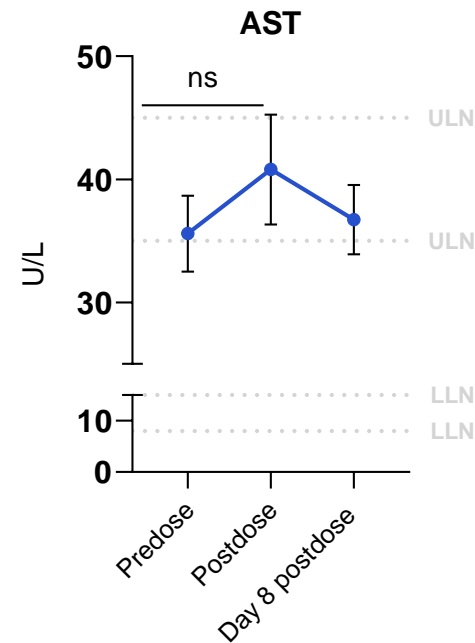

**B**

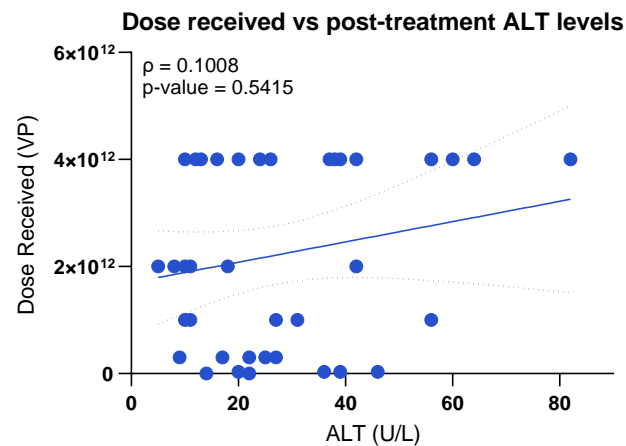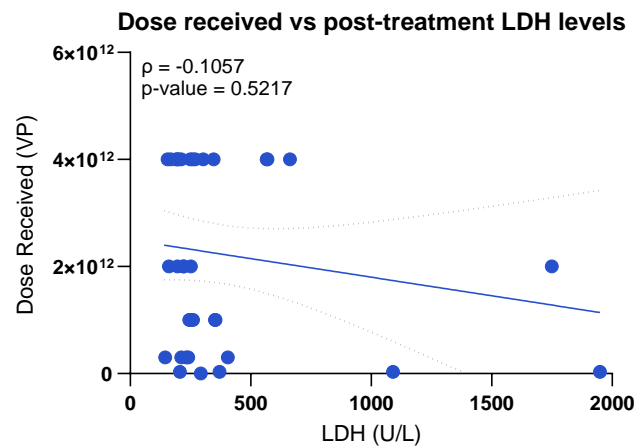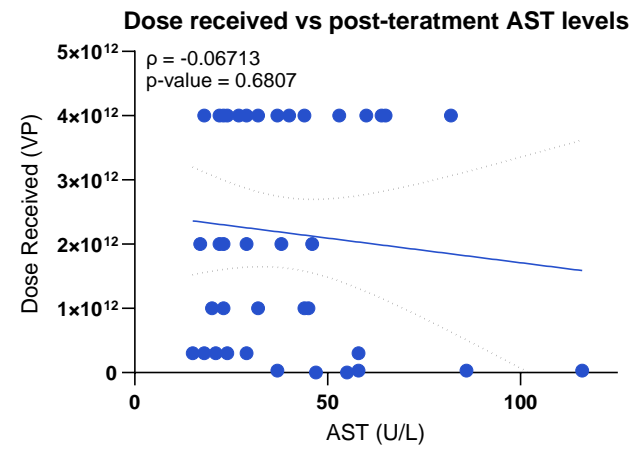

Supplement: Supplementary file 6 — Supplementary Material 6: Supplementary Fig. 1. Liver enzyme changes and correlation with treatment dose. A. Changes in liver enzymes alanine aminotransferase (ALT), lactate dehydrogenase (LDH), and aspartate transaminase (AST). Data are presented as mean ± SEM. Differences between timepoints were compared using Mann–Whitney U test where ns > 0.05. LLN = lower limit of normal. ULN = upper limit of normal. B. Spearman correlation of dose received and day 1 post-treatment liver enzyme levels. [file 13046_2024_3219_MOESM6_ESM.pdf]

# Supplementary Figure 2

**A**

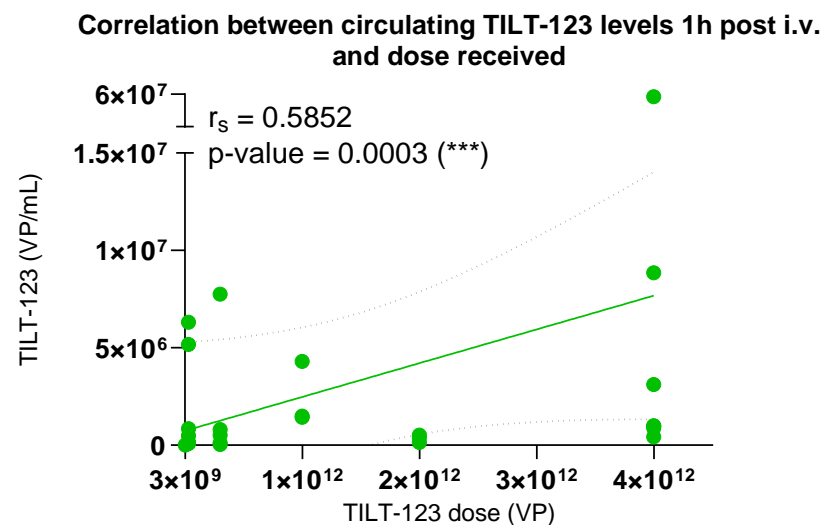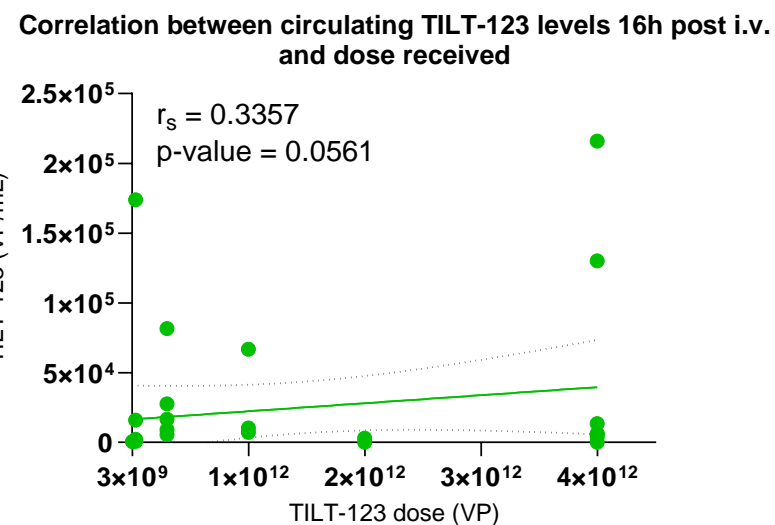

**B**

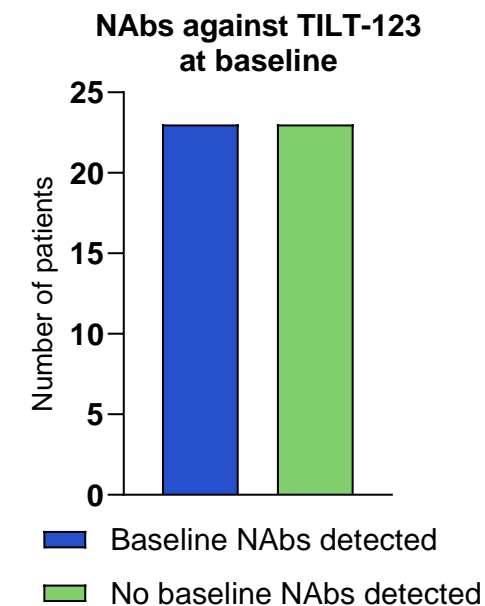

**C**

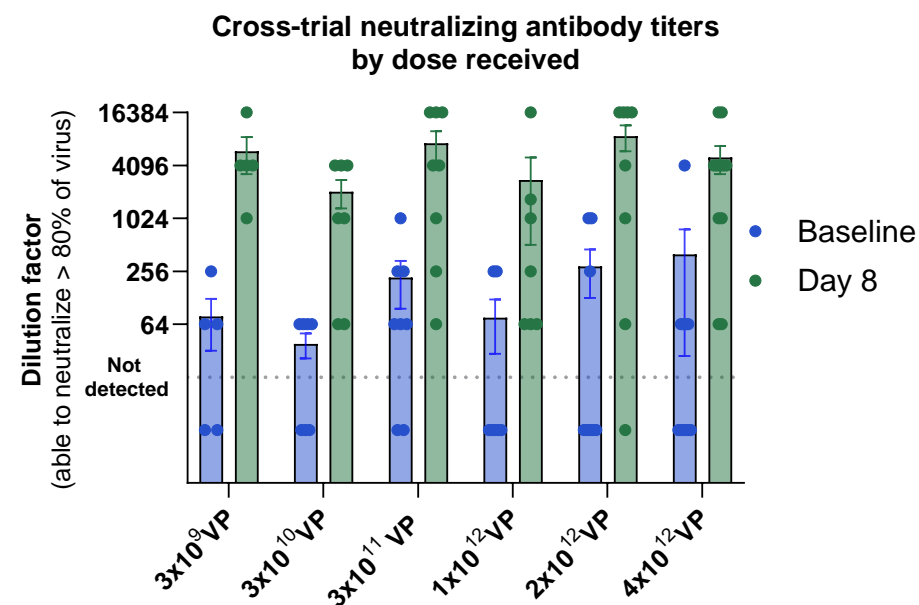

**D**

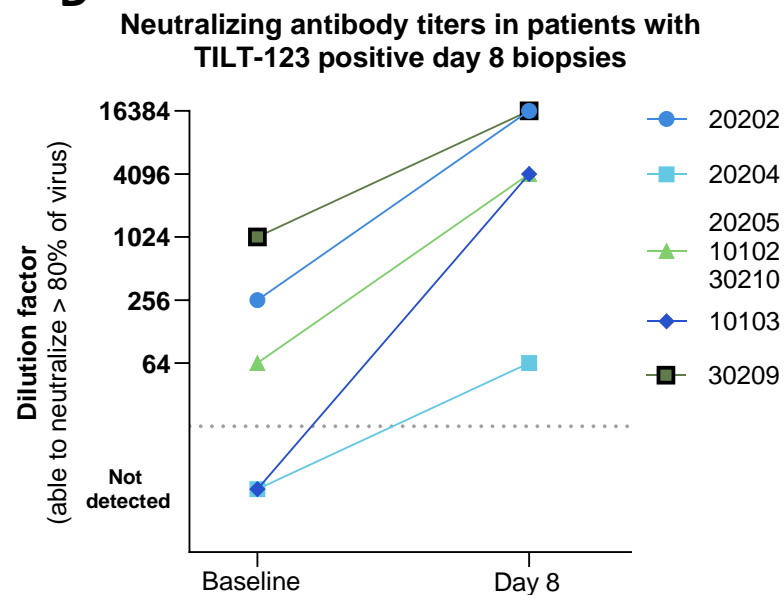

**E**

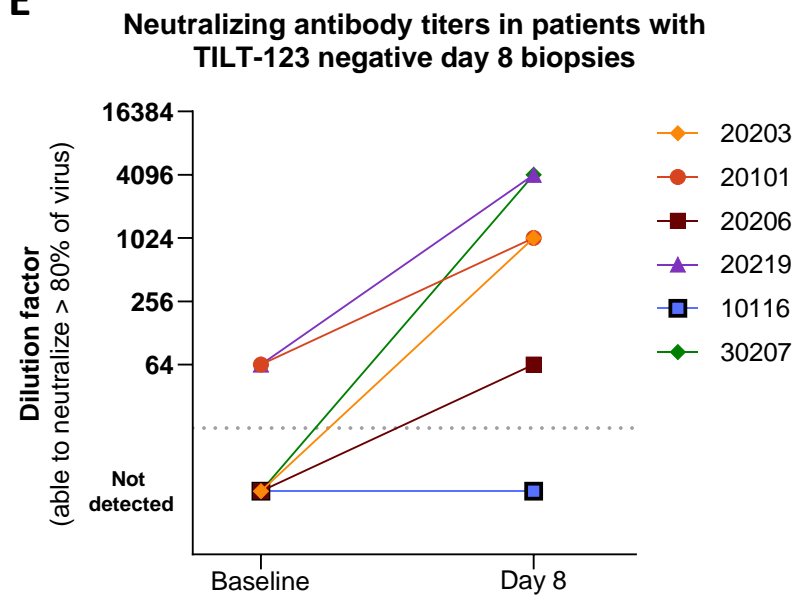

Supplement: Supplementary file 7 — Supplementary Material 7: Supplementary Fig. 2. Neutralizing antibody levels across trials. A. Spearman correlation analysis of circulating levels of TILT-123 1 h or 16 h post-injection and dose received where ***p < 0.001. B. Number of patients with NAbs detected or not detected at baseline. C. Cross-trial baseline and post-treatment neutralizing antibody titers, by dose received. Data are presented as mean ± SEM. D. NAb titers of patients with TILT-123 detected in day 8 biopsies. Patients 20202, 20204, and 20205 were enrolled in TUNIMO. Patients 10102, 10103 and 30209, 30210 were enrolled in TUNINTIL and PROTA trials, respectively. E. Neutralizing antibody titers in patients with TILT-123 negative day 8 biopsies. Patients 20203, 20101, 20206, 20219 were enrolled in TUNINTIL. Patients 10116 and 30207 were enrolled in TUNIMO and PROTA, respectively. [file 13046_2024_3219_MOESM7_ESM.pdf]
